# Short-Term In Vitro Exposure of Human Blood to 5G Network Frequencies: Do Sex and Frequency Additionally Affect Erythrocyte Morphometry?

**DOI:** 10.3390/biomedicines13020478

**Published:** 2025-02-15

**Authors:** Nikolino Žura, Silvijo Vince, Porin Perić, Marinko Vilić, Krešimir Malarić, Vladimira Rimac, Branka Golubić Ćepulić, Marina Vajdić, Ivan Jurak, Suzana Milinković Tur, Nina Poljičak Milas, Marko Samardžija, Jakob Nemir, Mirjana Telebuh, Ivona Žura Žaja

**Affiliations:** 1 Department of Physiotherapy, University of Applied Health Sciences, 10000 Zagreb, Croatia; nikolino.zura@zvu.hr (N.Ž.); ivan.jurak@zvu.hr (I.J.); mirjana.telebuh@zvu.hr (M.T.); 2 Department of Rheumatology and Rehabilitation, University Hospital Center Zagreb, 10000 Zagreb, Croatia; marina.vajdic@kbc-zagreb.hr; 3 Clinic of Obstetrics and Reproduction, Faculty of Veterinary Medicine, University of Zagreb, 10000 Zagreb, Croatia; svince@vef.unizg.hr (S.V.); smarko@vef.unizg.hr (M.S.); 4 Department of Physical Medicine and General Rehabilitation, Faculty of Medicine, University of Zagreb, 10000 Zagreb, Croatia; 5 Department of Physiology and Radiobiology, Faculty of Veterinary Medicine, University of Zagreb, 10000 Zagreb, Croatia; mvilic@vef.unizg.hr (M.V.); tur@vef.unizg.hr (S.M.T.); 6 Department of Communication and Space Technologies, Faculty of Electrical Engineering and Computing, University of Zagreb, 10000 Zagreb, Croatia; kresimir.malaric@fer.hr; 7 Department of Transfusion Medicine and Transplantation Biology, University Hospital Center Zagreb, 10000 Zagreb, Croatia; vladimira.rimac@kbc-zagreb.hr (V.R.); branka.golubic.cepulic@kbc-zagreb.hr (B.G.Ć.); 8 Department of Pathophysiology, Faculty of Veterinary Medicine, University of Zagreb, 10000 Zagreb, Croatia; nmilas@vef.unizg.hr; 9 Department of Neurosurgery, University Hospital Center Zagreb, 10000 Zagreb, Croatia; jakob.nemir@kbc-zagreb.hr

**Keywords:** 5G electromagnetic radiation, 5G network frequencies, in vitro exposure, human blood, haematological parameters, erythrocyte morphometry, erythrocyte subpopulations

## Abstract

**Background/Objectives**: This study assessed the effects of 5G radiofrequency electromagnetic radiation (RF-EMR) at different frequencies (700 MHz, 2500 MHz, 3500 MHz) on the complete blood count (CBC), erythrocyte morphometry, and platelet activation after the short-term in vitro exposure of human blood. **Methods**: Blood samples from 30 healthy volunteers (15 men and 15 women, aged 25–40 years old) were collected at three intervals (14 days apart). For each collection, four tubes of blood were drawn per volunteer—two experimental and two controls. Experimental samples were exposed to 5G RF-EMR for 2 h at room temperature using a half-cone gigahertz transverse electromagnetic cell. The CBC was analysed via a haematology analyser, the erythrocyte morphometry was analysed using the SFORM program, and platelet activation was analysed via flow cytometry. **Results**: The CBC and platelet activation showed no significant differences between the experimental and control samples. However, the erythrocyte morphometry exhibited notable changes. At 700 MHz, the erythrocyte size, contour, and membrane roughness increased significantly for both sexes, with women’s cells showing greater sensitivity. At 2500 MHz, women exhibited an increased contour index and a decreased solidity and form factor. At 3500 MHz, women showed an increased contour index and outline but a decreased solidity, elongation, and form factor. Cluster analysis identified two erythrocyte subpopulations: smaller, rounder cells with smooth membranes and larger cells with rougher membranes. **Conclusions**: These results indicate that 5G RF-EMR exposure significantly alters erythrocyte morphometry. The strongest effects were observed at 700 MHz, where men exhibited greater membrane roughness, and women showed larger and rounder erythrocytes. These findings suggest that short-term in vitro 5G RF-EMR exposure disrupts the cytoskeleton, increasing membrane permeability and deformability.

## 1. Introduction

The widespread use of radiofrequency electromagnetic radiation (RF-EMR), particularly with the introduction of 5G networks, has raised concerns about its potential effects on human and animal health [1,2,3]. Due to the lack of long-term studies, further research is needed to better understand these potential health effects [2,3,4]. Previous studies have suggested that RF-EMR affects genotoxicity, cell proliferation, gene expression, cell membrane function, and the immune, haematopoietic, and reproductive systems [4,5,6]. However, data on the effects of 5G RF-EMR on blood cells are limited, as 5G uses different frequencies and technology compared to earlier generations of mobile networks.

RF-EMR applications have been steadily increasing, particularly in telecommunications and even in clinical hospital settings [7]. Mobile communication sources exert biological effects through non-thermal RF-EMR mechanisms [8]. Healthcare workers handling blood samples, as well as patients using mobile devices, may additionally expose blood samples to RF-EMR, potentially affecting the quality of these samples and influencing diagnostic results, such as complete blood count (CBC) analyses. Blood products like erythrocyte and platelet concentrates might also be affected during production, storage, and handling, potentially reducing their lifespan or functionality by altering their morphology, which is significant for transfusion medicine [5,9].

RF-EMR is known to damage cellular organelles, such as cell membranes, mitochondria, and DNA [10]. Following RF-EMR exposure, cell membranes undergo “shock,” causing electroporation, during which aquaporins form, disrupting ion balance and altering cell shape [11,12]. RF-EMR also triggers cell necrosis, apoptosis, or autophagy [10], alters membrane receptor function [13,14], and leads to an overproduction of free radicals [4,15,16], which weakens the cell’s antioxidant defences, promoting oxidative stress [17].

Oxidative stress caused by RF-EMR results in the release of excess reactive oxygen species (ROS) from the mitochondria, damaging the erythrocyte membrane and reducing deformability [12,18,19]. This accelerates ageing and induces early eryptosis [20,21]. Changes in cell shape due to the loss of deformability can also result from pathological conditions [22], ROS accumulation, or natural ageing [23]. Erythrocyte shape is influenced by cytoskeletal properties and adenosine triphosphate (ATP) levels, with lower ATP causing cells to shift from a disc to an echinocyte form [24,25]. For instance, sickle-shaped erythrocytes show elevated oxidative stress and reduced ATP levels [26,27].

Erythrocyte deformability refers to their ability to change shape in response to applied force, allowing them to pass through narrow blood vessels and deliver oxygen to tissues, crucial for ATP generation [13]. Factors like nitric oxide (NO) influence deformability, which is reduced at lower NO levels and increased at higher NO levels [28,29,30]. Oestradiol also enhances erythrocyte deformability by increasing CuZn superoxide dismutase activity and NO production, helping maintain oxidative balance [30,31,32,33,34].

Morphometry, the simplest form of image cytometry, refers to the measurement of cellular features in two-dimensional images [35]. Morphometric analysis has been used to evaluate the effects of RF-EMR on boar sperm morphology after short-term in vitro exposure [36].

The literature lacks data on the effects of 5G RF-EMR at different frequencies on CBC parameters, erythrocyte morphometry, and platelet activation following in vivo or in vitro exposure. This study hypothesises that RF-EMR from 5G networks causes changes in the CBC levels, erythrocyte morphometry, and platelet activation in human blood after short-term in vitro exposure, with gender-dependent effects. Therefore, this study aims to assess the impact of 5G RF-EMR at 700 MHz, 2500 MHz, and 3500 MHz on CBC levels, erythrocyte morphometry, and platelet activation following the short-term in vitro exposure of blood from male and female volunteers.

## 2. Materials and Methods

### 2.1. Ethics and Welfare Approval Statement

This study was conducted in accordance with the Declaration of Helsinki and was approved by the Ethics Committee of the Clinical Hospital Centre Zagreb, Croatia (record no. 8.1-22/99-2; file no. 02/013 AG); approval date: 4 April 2022.

### 2.2. Blood Collection

Blood samples were taken from 30 clinically healthy volunteers (15 women and 15 men), employees of the Clinical Hospital Centre Zagreb, Croatia, aged 25 to 40 years old. Blood collection from the volunteers was performed by an individual with a Master of Nursing degree, authorised to work independently, according to the standard procedure in the morning hours (around 9:00 a.m.). To minimise the potential impact of individual variability (diet, lifestyle, psychological stress, and health conditions between blood collections) on baseline values and experimental outcomes, a self-controlled study design was implemented. Each experimental sample had its own control sample, with both collected simultaneously from the same individual. Volunteer blood was collected three times at 14-day intervals. Four tubes of blood were drawn from each volunteer each time: two tubes with ethylenediaminetetraacetic acid (EDTA, Greiner Bio-One International GmbH, Kremsmünster, Austria) anticoagulant and two tubes with sodium citrate (3.8%) (total 8 mL, 4 × 2 mL).

### 2.3. Exposure of Blood Samples to 5G Radiofrequency Electromagnetic Radiation Under Laboratory Conditions

**Figure 1:**
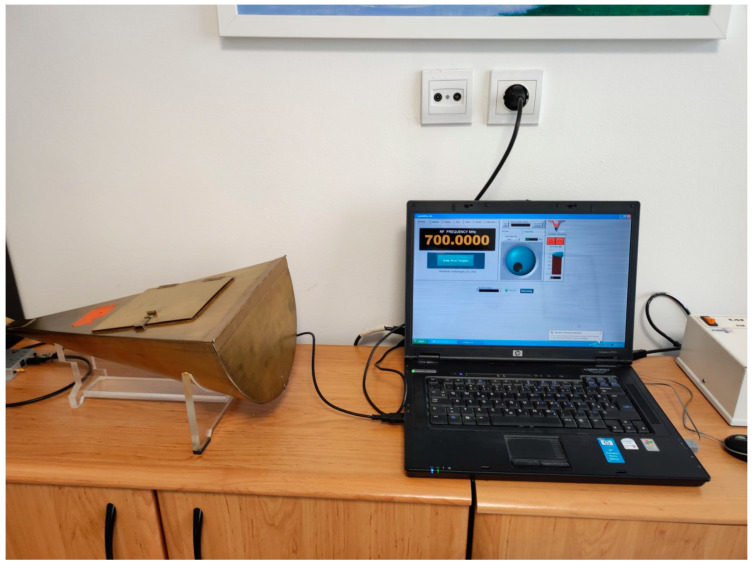
Half-cone gigahertz transversal electromagnetic cell and signal generator operated by a laptop.

Each volunteer had their blood drawn on three separate occasions, with a two-week interval between each blood draw. On each occasion, four tubes of blood were collected, for a total of twelve tubes per volunteer over the course of four weeks. Immediately after collection, two of the tubes (one containing EDTA as an anticoagulant and the other containing sodium citrate at 3.8%) were exposed to continuous 5G RF-EMR. The blood was exposed to a single frequency (either 700 MHz, 2500 MHz, or 3500 MHz), with each frequency used on a different occasion, using freshly drawn blood. These frequencies are among those most commonly used for 5G communication, making them particularly relevant for investigating potential biological effects. The exposure was conducted in a half-cone gigahertz transversal electromagnetic (HCTEM) cell, forming the experimental group. The other two tubes from each volunteer were kept under the same conditions, in a metal box made of the same material as the HCTEM cell and located in the same room, but without exposure to 5G RF-EMR (control group). The experimental blood samples were exposed for 2 h at room temperature to three frequencies of continuous 5G RF-EMR (each frequency at a separate time) with an electric field strength of 10 V/m in the HCTEM cell, at the Department of Communication and Space Technologies, Faculty of Electrical Engineering and Computing, University of Zagreb, Zagreb, Croatia (Figure 1) [36]. The HCTEM enabled precise control of the electric field strength at specific 5G frequencies, thereby improving the reliability and reproducibility of this study’s findings.

In both the experimental and control human whole blood samples, CBC indicators and platelet activation were determined. All analyses were performed in duplicate, meaning that each sample was analysed twice to ensure the accuracy and reliability of the results. Prior to analysis for CBC indicators, the blood samples were mixed in automatic mixers, and immediately before being placed into the device, the samples were mixed by inverting the tubes approximately 10 times at room temperature (≈20 °C). This approach aligns with standard laboratory practices, where blood samples are typically analysed at room temperature within a specific timeframe post collection. CBC indicators and platelet activation were assessed within 2 h and 30 min after blood collection. Due to the sensitivity of the assays for assessing platelet activation, samples had to be analysed as soon as possible after exposure, ideally within 30 min, and no later than 2 h and 30 min post collection to ensure reliable results.

### 2.4. Analysis of Complete Blood Count Indicators

Complete blood count indicators were determined at the Department of Transfusion Medicine and Transplantation Biology, Clinical Hospital Centre Zagreb, using an Advia 2120i haematology analyser (Siemens Healthcare Diagnostics, Marburg, Germany), with corresponding reagents from the same manufacturer. All CBC analyses (a total of 180 whole blood samples, i.e., 2340 individual analyses) were performed on whole blood collected in tubes containing the anticoagulant EDTA. The CBC indicators included the following: total number of leukocytes and their subpopulations—neutrophil, eosinophil, and basophil granulocytes, lymphocytes, and monocytes—expressed in both relative and absolute values; number of erythrocytes; reticulocytes; haemoglobin concentration; haematocrit; mean cell volume (MCV); mean cell haemoglobin (MCH); mean cell haemoglobin concentration (MCHC); number of platelets; and mean platelet volume (MPV).

### 2.5. Analysis of Platelet Activation

**Figure 2:**
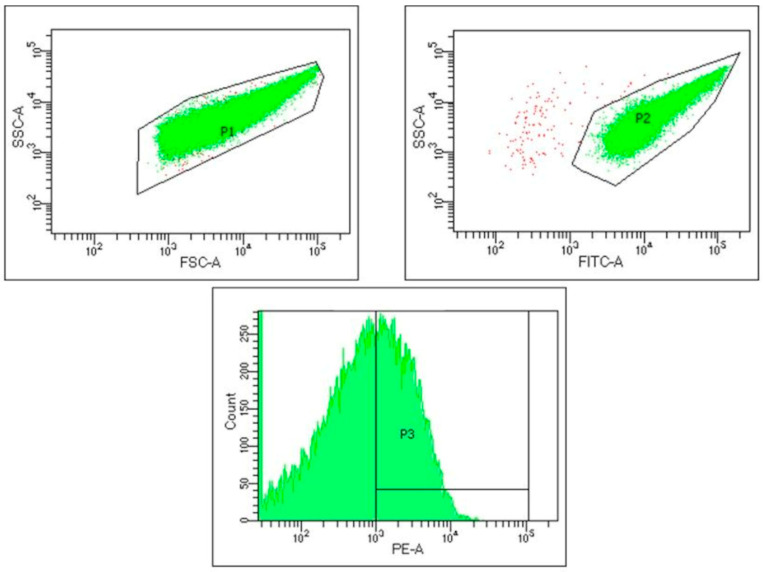
Illustration of the protocol used for platelet activation analysis. FSC–forward scatter; SSC—side scatter; FITC—fluorescein isothiocyanate; PE—phycoerythrin. In P1, platelets are gated by size and granularity, while in P2, platelets are gated using anti-CD41a-FITC antibody. P3 presents platelets activated using anti-CD62-PE antibody.

The analysis of platelet activation using the flow cytometry method was conducted at the Department of Transfusion Medicine and Transplantation Biology, Clinical Hospital Centre Zagreb, on a BD FACS Canto II flow cytometer (BD Biosciences, San Jose, CA, USA). Platelet activation was determined as part of this analysis. The first step involved isolating platelets from the volunteers’ whole blood collected in test tubes containing 3.8% sodium citrate. The samples were centrifuged at 120× *g* for 5 min to obtain platelet-rich plasma (PRP). After centrifugation, 40 µL PRP and 20 µL antibody were added to the test tubes. The monoclonal antibodies used in this study were anti-CD41-FITC to label all platelets in the sample (BD Pharmingen, FITC Mouse Anti-HUMAN CD41a; BD Biosciences, San Jose, CA, USA) and anti-CD62P-PE (BD Biosciences, San Jose, CA, USA) to identify activated platelets. After labelling, the platelets were incubated with the antibodies for 5 min in the dark at 37 °C. All samples were labelled and analysed in duplicate. BD FACS Diva software (Version 8.0.1; BD Biosciences, San Jose, CA, USA) was used for the analysis according to a predefined protocol (Figure 2).

### 2.6. Morphometric Analysis of Erythrocytes

**Figure 3:**
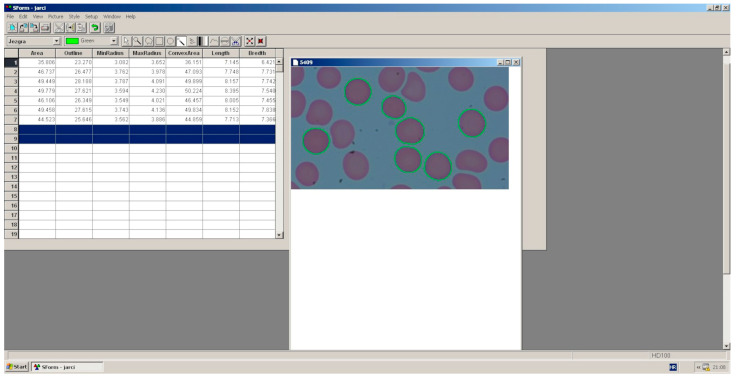
Morphometric analysis of human erythrocytes on a personal computer using the SFORM program (VAMSTEC, Zagreb, Croatia).

Blood smears were prepared from the human blood samples (both experimental and control groups) and stained using Pappenheim’s method (May–Grünwald and Giemsa solutions). The stained blood smears were then subjected to computer analysis (basic morphometric characteristics of erythrocytes) using the SFORM program (Version 1.0; VAMSTEC, Zagreb, Croatia) (Figure 3). The system includes a high-resolution colour camera (Donpisha 3CCD), which digitises the image under a 100× magnification lens of the Olympus BX 41 light microscope and transfers it to a personal computer. The primary (size) morphometric indicators and erythrocyte shape indicators were then determined. A total of 180 stained blood smears of human erythrocytes were analysed, with more than 100 erythrocytes measured for each smear. A total of 26,068 erythrocytes were measured from the control and experimental smears. The erythrocytes were measured in 90 control smears and 90 experimental smears. Only erythrocytes that were not overlapping with others were analysed. The boundaries of the erythrocyte cytoplasm were marked interactively (initially using an automatic erythrocyte rounding command) and then manually corrected with a computer mouse. The following parameters were determined for the erythrocyte cytoplasm: area (μm^2^), outline (μm), convex area or convexity (μm^2^), minimum and maximum radius (μm), length (μm), and width (μm). The shape indicators for erythrocytes were calculated based on primary indicators (length, width, area, and outline) using the following formulae:

(1) $$roundess=\frac{4\times a}{\pi \times {\left(maximum\;radius\right)}^{2}}$$

(2) $$rugosity=4\pi \times \frac{area}{{outline}^{2}}$$

(3) $$ellipticity=\frac{length}{width}$$

(4) $$elongation=\frac{length-width}{length+width}$$

(5) $$solidity=\frac{area}{convex\;area}$$

(6) $$contour\;index=\frac{outline}{\sqrt{area}}$$

### 2.7. Statistical Data Processing

Statistical analysis was performed using the SAS 9.4 software package (Statistical Analysis Software 2002–2012, SAS Institute Inc., Cary, NC, USA).

Descriptive statistics were performed using the PROC MEANS and PROC FREQ modules. The normal distribution of the data was tested using the PROC TRANSREG module. If the assumptions of normal distribution of the dependent variables were violated, and in the case of heterogeneity of variances, the transformation of variables was performed using the BOX-COX transformation, usually by logarithmic or exponential transformation.

The main analysis model was conducted using the GLIMMIX procedure and included the fixed effect of the group. The results are presented as least squares means (LSMs) and 95% confidence intervals. The Tukey–Kramer method of multiple comparisons at the level of statistical significance *p* < 0.05 was used to compare mean values. If a transformation was performed after the analysis, the data were transformed back to the original values and are presented as such in the tables and figures.

In addition to analysing the values of each morphometric indicator individually, multivariate data clustering analyses (CLUSTERS) were also performed in several steps to obtain erythrocyte subpopulations (ESs) based on the data of the main morphometric indicators. The first analysis performed was the principal components analysis to obtain the characteristic values (eigenvalues) of the morphometric indicators using the Kaiser criterion (λ ≥ 1) to determine the number of principal components. The second analysis was performed to obtain the number of clusters (subpopulation) with the HPCLUS procedure using the aligned box criterion value. The last analysis was conducted to obtain well-separated clusters with non-random initialisation with the FASTCLUS procedure and to perform step-wise discriminant analysis with the STEPDISC procedure. To solve the problem of outliers, the FASTCLUS procedure was performed using the LEAST and STRICT option. To test for differences in the distribution of ESs between the groups, the chi-square test was performed.

## 3. Results

### 3.1. Complete Blood Count Indicators and Platelet Activation Depending on the Frequency of 5G Radiofrequency Electromagnetic Radiation and Sex

Table 1, Table 2, Table 3, Table 4 and Table 5 show the CBC indicators in humans, of both sexes, of the three independent experimental groups (exposed to 5G RF-EMR at a frequency of 700 MHz, 2500 MHz, or 3500 MHz) and control groups (non-exposed samples).

Table 1, Table 2, Table 3, Table 4 and Table 5 show that the mean values of the experimental and control samples differed significantly for four haematological indicators. Specifically, the haemoglobin concentration was significantly higher (*p* < 0.05) in the experimental group exposed to 3500 MHz; the lymphocyte count was significantly higher (*p* < 0.05) in women in the experimental group exposed to 2500 MHz; the MCV was significantly lower (*p* < 0.05) in men in the experimental group exposed to 2500 MHz; and the MPV was significantly lower (*p* < 0.05) in women in the experimental group exposed to 3500 MHz.

### 3.2. Morphometric Indicators of the Size and Shape of Erythrocytes Depending on the Frequency of 5G Radiofrequency Electromagnetic Radiation and Gender

Table 6 and Table 7 show the morphometric size and shape indicators of human erythrocytes, of both sexes, of the three independent experimental groups (exposed to 5G RF-EMR at frequency of 700 MHz, 2500 MHz, and 3500 MHz) and control groups (non-exposed samples).

The values of the area, outline, minimum and maximum radius, convex area, length, width, and contour index in the experimental group of samples exposed to a frequency of 700 MHz were significantly higher (*p* < 0.001), while the values of the solidity and form factor were significantly lower (*p* < 0.001) in both men and women. However, the differences between the control and experimental samples in most indicators (area, outline, minimum and maximum radius, convex area, length, width) were more statistically significant in women than in men, while the differences in the contour index, solidity, and form factor in the control and experimental samples exposed to a frequency of 700 MHz were less statistically significant in women compared to men.

In the experimental samples exposed to a frequency of 2500 MHz, significantly higher values were recorded for the erythrocyte maximum radius (*p* < 0.01), outline, and contour index at the level of statistical significance (*p* < 0.001) and the maximum radius, while a significantly lower value was found for the solidity (*p* < 0.01), roundness (*p* < 0.01), and form factor (*p* < 0.001), regardless of sex. In women, significantly higher values were recorded for the contour index (*p* < 0.001), and significantly lower values for the solidity (*p* < 0.01) and form factor (*p* < 0.001) were found in experimental samples exposed to 2500 MHz.

A significantly higher value for the erythrocyte outline and contour index was recorded (*p* < 0.001) in the experimental samples exposed to a frequency of 3500 MHz, while a significantly lower value for the solidity and form factor (*p* < 0.001) was found in women. In addition, significantly lower values for the ellipticity (*p* < 0.01) and elongation of erythrocytes (*p* < 0.05) were found in women in the experimental samples exposed to a frequency of 3500 MHz.

### 3.3. Distribution of Subpopulations of Erythrocytes in Groups Categorised According to the Morphometric Indicators

When analysing the principal components before grouping the human erythrocytes, three components with a characteristic value (λ≥ 1) were retained. All three components together explained 97% of the variance in the morphometric indicators of human erythrocyte size and shape (Table 8).

**Figure 4:**
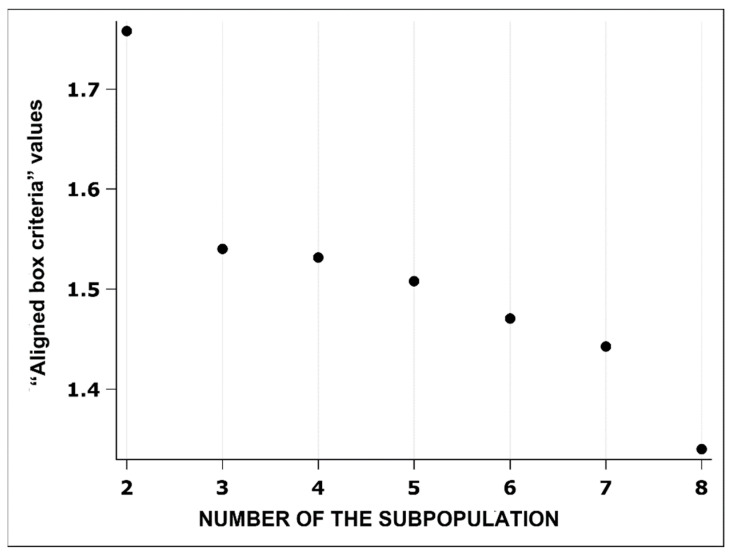
Optimal number of subpopulations based on the highest value of the aligned box criteria.

The first factor was the erythrocyte size (outline, convex area, area, length and width), and the most important value for this factor was the erythrocyte outline. The second and third factors focused on the erythrocyte shape (roundness, form factor, contour index, elongation, ellipticity, and solidity), for which elongation was the most important variable for the second factor, while solidity was the most important variable for the third factor (Table 8). The final number of ESs was determined using the value of the “aligned box criteria”. This analysis showed that the two subpopulations are the most optimal, as the “aligned box criteria” value is the highest (Figure 4).

Cluster analysis resulted in two well-defined subpopulations of erythrocytes based on morphometric indicators of size and shape (Table 9). The first subpopulation of erythrocytes (ES 1) consisted of smaller and rounder erythrocytes with more regular/smoother membranes (67.5%), while the second subpopulation (ES 2) consisted of larger erythrocytes with more irregular/rough membranes (32.5%).

**Figure 5:**
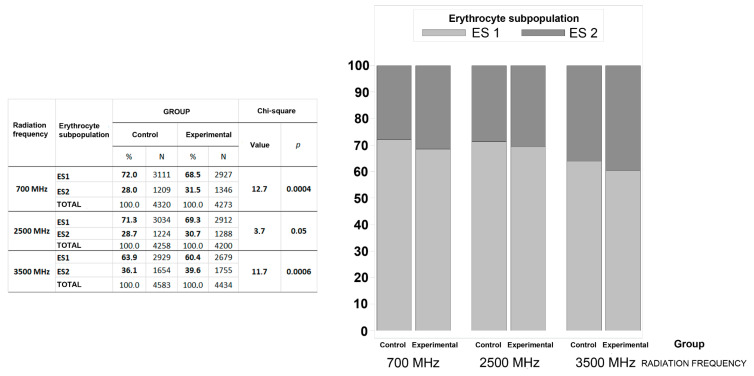
Proportion of erythrocyte subpopulations (ESs) of humans in the control and experimental groups depending on the frequency of 5G radiofrequency electromagnetic radiation (700 MHz, 2500 MHz, or 3500 MHz). ES 1—erythrocyte subpopulation with smaller and rounder erythrocytes and with more regular/smoother membranes; ES 2—erythrocyte subpopulation with larger erythrocytes and with more irregular membranes.

Figure 5 indicates that the experimental group exposed to 700 MHz showed a higher percentage of erythrocytes in the ES2 subpopulation (31.5 vs. 28.0%) and a lower percentage in the ES1 subpopulation (68.5 vs. 72.0%) compared to the control group (*p* = 0.0004). The experimental group exposed to 2500 MHz had a higher proportion of erythrocytes in the ES2 subpopulation (30.7 vs. 28.7%) and a lower proportion in the ES1 subpopulation (69.3 vs. 71.3%) compared to the control group (*p* = 0.05). The experimental group exposed to 3500 MHz had a significantly higher (*p* = 0.0006) proportion of erythrocytes in the ES2 subpopulation (39.6 vs. 36.1%) and a significantly lower proportion in the ES1 subpopulation (60.4 vs. 63.9%) compared to the control group.

## 4. Discussion

In this study, it was found that 5G RF-EMR at the investigated frequencies (700 MHz, 2500 MHz, and 3500 MHz) did not affect the CBC parameters or platelet activation in human blood following short-term (2 h) in vitro exposure but had an impact on the morphometry of human erythrocytes. In the available literature, there are no data on the investigated effect of different frequencies, especially of 5G electromagnetic radiation, on CBC parameters, human erythrocyte morphometry, and platelet activation following the in vivo or in vitro exposure of humans or animals or their blood. The only available data on human blood samples exposed in vitro to RF-EMR from 3G and 4G technology on haematology parameters are from studies conducted by Kumari et al. [37] and Christopher et al. [38]. Kumari et al. [37] found a significant reduction in the number of erythrocytes, leukocytes, and platelets following the exposure of volunteer blood in vitro to continuous RF-EMR generated by a mobile phone (dual-band EGSM at 900/1800 MHz) for a duration of 1 h. Christopher et al. [38] found that the exposure of human blood samples for 1 h to continuous RF-EMR generated by a 4G mobile phone operating in the transmission frequency range of 2.3 to 2.4 GHz led to a significant decrease in the platelet count and an increase in the leukocyte count and haemoglobin concentration. The results of this study contradict those by Kumari et al. [37] and Christopher et al. [38], and the possible reason for this is the use of different analytical procedures, i.e., the use of different mobile technology/generation, the duration, and the frequency to which the blood samples were exposed.

In this study, 5G RF-EMR at 700 MHz, 2500 MHz, and 3500 MHz did not affect platelet activation in human blood exposed in vitro. The effect of RF-EMR on human platelets was investigated by Lippi et al. [39], whose findings are partially consistent with the results presented here. Specifically, the authors observed a significant reduction in platelet aggregation/adhesion (platelet activation) and an increase in platelet size (increase in MPV value) following the in vitro exposure of human blood to RF-EMR from a mobile phone at a frequency of 900 MHz (3G network) for 30 min. However, the method for determining platelet activation differed; Lippi et al. [39] used a platelet function analyser (PFA-100), a functional method using whole blood, while this study used flow cytometry and platelet-rich plasma samples. The difference between these two methods and the matrix may have influenced the results, making them less comparable. Furthermore, Lippi et al. [39] used the same haematology device to detect the platelet count and MPV as was used in this study. They did not observe a significant change in the platelet count following the RF-EMR exposure of human blood samples, which was also found in this study. However, they reported an increase in the MPV value, whereas in this study, the only change found in the MPV was a significantly lower value in women in the experimental group exposed to 3500 MHz. It is likely that the difference in the results obtained between this study and Lippi et al. [39] was caused by the use of different mobile technologies and a different radiation frequency, which likely has a more significant effect on the activation of human platelets, consequently leading to their enlargement.

In this study, blood samples from both male and female volunteers were included, and the effect of 5G RF-EMR on erythrocyte morphometry was additionally investigated, taking sex into account. The effect of 5G RF-EMR on human erythrocytes from both male and female volunteers, short-term-exposed in vitro, manifested as significant enlargement, increased roundness, and roughness of the erythrocyte membranes. The only available study that involved in vitro exposure to RF-EMR was conducted by Nguyen et al. [5], in which rabbit erythrocytes were exposed three times to an 18 GHz frequency for 1 min each. Their study demonstrated that rabbit erythrocytes exposed to this electromagnetic field exhibited increased membrane permeability without compromising cell viability. Notably, the membrane permeability returned to its original state after approximately 10 min, suggesting a reversible effect. It is important to note that Nguyen et al.’s study involved significantly shorter exposure durations (three exposures of 1 min each at 18 GHz) and different protocols compared to ours. Additionally, it did not focus on 5G RF-EMR at varying frequencies. Given these differences in exposure parameters, it is not possible to directly infer the reversibility of the erythrocyte morphometric changes observed in our study. In this study, significant changes in erythrocyte morphometry were observed under short-term exposure to 5G RF-EMR; however, it remains unclear whether these changes are transient and reversible. To determine this definitively, further research incorporating extended post-exposure observation periods and varying exposure conditions is necessary. Nguyen et al. [5] also suggested that erythrocyte membranes become permeable due to mechanical disruption caused by RF-EMR exposure. Although those authors used a much higher frequency for in vitro erythrocyte exposure (18 GHz), applied three times for only one minute, whereas much lower frequencies (0.7 GHz, 2.5 GHz, and 3.5 GHz) were used in this study, the observed effect on erythrocytes appears to be similar, namely, an increase in membrane permeability and shape alteration due to mechanical disruption. Mechanical disturbances induced by RF-EMR can increase cell membrane permeability [5]. Furthermore, Girasole et al. [40] stated that one of the mechanisms leading to the enlargement and increased roundness of erythrocytes is the weakening of the cytoskeletal structure. Changes in the cytoskeleton structure can disrupt the cell membrane, making the cells more permeable and susceptible to membrane deformation. The transformation from a biconcave (thermodynamically more favourable) to a spherical shape [41], besides being a consequence of erythrocyte ageing, can also result from conformational changes in membrane proteins and the redistribution of membrane phospholipids. This leads to the separation of one phospholipid layer from the other, causing a shape change [27,42,43,44] and the formation of convex structures on the cell surface (e.g., echinocyte protrusions/roughness) [45]. Under physiological conditions, normal human erythrocytes have a biconcave disc shape, and it is known that various agents can alter their shape. For example, increased extracellular fluid osmolality, high pH, decreased ATP, etc., lead to wrinkled or roughened membrane shapes, known as echinocytes, characterised by an increased convex surface and the presence of “spikes” or roughness. Based on all of the above, it seems that RF-EMR in in vitro-exposed human erythrocytes leads to changes in their shape and cell membrane by a similar mechanism, with the excessive production of ROS causing oxidative stress [46].

In this study, the negative effect of 5G RF-EMR on the morphometry of human erythrocytes in in vitro-exposed blood depended on the radiation frequency and sex. The most harmful effect on the morphometry of human erythrocytes was observed following exposure to radiation at a frequency of 700 MHz. The obtained results can be interpreted in light of the fact that the effects of RF-EMR on bodily systems and cells depend on the frequency, field strength, and duration of exposure [47,48,49,50]. This study found that women’s erythrocytes were more susceptible to the in vitro effects of 5G RF-EMR. Specifically, exposure at a frequency of 700 MHz had a more significant impact on the morphometric values of women’s erythrocytes compared to those of men, while significant effects of exposure at 3500 MHz and 2500 MHz on erythrocyte morphometry were observed only in women. Moreover, erythrocytes in men exhibited more pronounced roughness, whereas erythrocytes in women showed greater enlargement and increased roundness, especially following exposure to 5G RF-EMR at 700 MHz. Women’s erythrocytes have greater “deformability” compared to men’s erythrocytes [30,31,32,34], which may explain the results obtained. Specifically, the erythrocyte membranes in men became rougher compared to those in women, while in women, the membranes exhibited greater enlargement and roundness, likely due to the higher elasticity of women’s erythrocyte membranes. In men’s erythrocytes, the membranes roughened more significantly, probably due to more pronounced conformational changes in the proteins and changes in the lipids of the cell membrane.

The obtained results in this study, showing that women’s erythrocytes are more susceptible to significant changes in morphometric parameters following short-term in vitro exposure to 5G RF-EMR, are difficult to explain based on known facts. It has been proven that men’s erythrocytes experience accelerated haemolysis during storage due to osmotic or oxidative stress observed after transfusion, compared to women’s erythrocytes [51,52,53]. The sex difference in erythrocyte haemolysis after transfusion is related to the properties of the erythrocyte membranes themselves and is not mediated by factors in blood plasma or female sex hormones. Testosterone promotes sex differences, specifically increased sensitivity to erythrocyte haemolysis, likely through a mechanism occurring during erythropoiesis that ultimately modulates the fragility and rheological properties of differentiated erythrocytes in men and male mice [53]. The results of this study suggest that women’s erythrocytes are more susceptible to the in vitro effects of 5G RF-EMR compared to men’s erythrocytes and can be interpreted in light of the fact that women’s erythrocytes exhibit greater “deformability” and respond more rapidly to external stress (RF-EMR exposure), resulting in more pronounced changes in membrane properties and a consequent increase in their size. This does not necessarily mean that this increase in erythrocyte size in women, compared to men, negatively affects their viability or functionality. Although in vitro-exposed erythrocytes in men showed less significant changes than in women, considering that they are more prone to faster haemolysis and have less deformability than women’s erythrocytes, there is a possibility that despite the less significant shape changes and more pronounced roughness, especially following exposure to 700 MHz, they would haemolyse faster than women’s erythrocytes.

The presence of ESs in humans, based on morphometric parameters and using the same statistical methods as in Žura Žaja et al. [54] on sheep, was also confirmed in this study following the in vitro exposure of blood to 5G RF-EMR at all investigated frequencies. A significant increase in the proportion of the erythrocyte subpopulation with larger erythrocytes and roughened membranes was observed compared to the proportion of the erythrocyte subpopulation with smaller, rounder erythrocytes with more regular, smoother membranes in human blood exposed in vitro. The main effect of erythrocyte ageing is associated with an increased proportion of larger erythrocytes with a roughened membrane [27]. Based on this, we can assume that the in vitro exposure of human blood to 5G RF-EMR accelerates the ageing of erythrocytes.

## 5. Conclusions

Exposure to 5G RF-EMR at the investigated frequencies does not affect CBC values or platelet activation in in vitro-exposed human blood, but it significantly alters erythrocyte morphometry. In vitro exposure to 5G RF-EMR led to increased size, roundness, and membrane roughness in erythrocytes from both male and female volunteers, with women’s erythrocytes being more susceptible. The most pronounced effect on erythrocyte morphometry was observed after exposure to 700 MHz. Men’s erythrocytes exhibited greater membrane roughness, while women’s erythrocytes showed a significant increase in size and roundness. These findings suggest that short-term in vitro 5G RF-EMR exposure disrupts the cytoskeleton, increasing membrane permeability and deformity. Further research should assess the impact of RF-EMR exposure on erythrocyte viability, function, and lifespan and potential sex-related differences. Investigating the temporal dynamics of erythrocyte responses is necessary to determine the reversibility of morphological changes. A deeper analysis of the physiological and biophysical mechanisms could further enhance the understanding of the effects of 5G radiation on blood cells.

## Figures and Tables

**Table 1:** Differential blood count indicators in humans of both sexes. Mean values and 95% confidence interval of three independent experimental groups (30 samples per group were exposed to 5G RF-EMR at a frequency of 700 MHz, 2500 MHz, and 3500 MHz) and control group.

		5G Radiofrequency Electromagnetic Radiation (5G RF-EMR) Frequencies					
		700 MHz		2500 MHz		3500 MHz	
Sample Size		30	30	30	30	30	30
Group	Gender	Control	Experimental	Control	Experimental	Control	Experimental
Neutrophils (%)	M	56.25 (52.25–60.17)	56.91 (52.92–60.82)	55.70 ^#^ (51.70–59.63)	55.91 ^#^ (51.91–59.84)	55.81 (51.81–59.43)	55.50 (51.50–59.43)
	F	58.37 (54.38–62.25)	58.21 (54.22–62.10)	61.73 ^#^ (57.77–65.73)	62.37 ^#^ (58.42–66.16)	59.90 (55.92–63.75)	59.75 (55.77–63.60)
	M+F	57.31 (54.49–60.09)	57.56 (54.75–60.33)	58.75 (55.93–61.51)	59.18 (56.37–61.93)	57.87 (55.05–60.63)	57.64 (54.82–60.41)
Basophils (%)	M	0.49 (0.39–0.61)	0.48 (0.38–0.60)	0.50 ^##^ (0.40–0.63)	0.55 ^##^ (0.45–0.68)	0.55 ^#^ (0.45–0.68)	0.55 ^#^ (0.45–0.68)
	F	0.35 (0.26–0.45)	0.36 (0.28–0.47)	0.30 ^##^ (0.22–0.40)	0.34 ^##^ (0.26–0.45)	0.39 ^#^ (0.31–0.51)	0.37 ^#^ (0.29–0.48)
	M+F	0.41 (0.34–0.49)	0.42 (0.35–0.50)	0.39 (0.32–0.47)	0.43 (0.36–0.52)	0.47 (0.40–0.55)	0.45 (0.38–0.54)
Eosinophils (%)	M	3.25 ^#^ (2.37–4.46)	3.25 ^#^ (2.36–4.45)	2.99 ^#^ (2.15–4.16)	3.03 ^#^ (2.18–4.20)	3.20 ^#^ (2.32–4.39)	3.09 ^#^ (2.23–4.26)
	F	1.84 ^#^ (1.20–2.81)	1.89 ^#^ (1.25–2.87^)^	1.53 ^#^ (0.96–2.44)	1.66 ^#^ (1.06–2.59)	1.71 ^#^ (1.10–2.65)	1.61 ^#^ (1.02–2.53)
	M+F	2.45 (1.88–3.19)	2.48 (1.91–3.22)	2.15 (1.61–2.85)	2.24 (1.70–2.96)	2.34 (1.79–3.07)	2.23 (1.69–2.94)
Lymphocytes (%)	M	33.95 (30.11–38.02)	33.75 (29.92–37.92)	34.31 ^#^ (30.45–38.38)	34.17 (30.32–38.24)	33.88 (30.04–37.95)	34.18 (30.33–38.25)
	F	33.79 (29.96–37.86)	33.96 (30.12–38.03)	28.83 ^#^ (25.19–32.76)	30.71 (26.99–34.70)	32.77 (28.97–36.81)	32.81 (29.00–36.85)
	M+F	33.87 (31.13–36.73)	33.86 (31.12–36.71)	31.50 (28.81–34.32)	32.42 (29.71–35.26)	33.32 (30.59–36.17)	33.49 (30.76–36.34)
Monocytes (%)	M	6.07 (5.29–6.95)	5.79 (5.03–6.66)	6.47 ^#^ (5.67–7.38)	6.33 ^##^ (5.53–7.23)	6.59 ^#^ (5.78–7.50)	6.65 ^#^ (5.84–7.57)
	F	5.63 (4.89–6.49)	5.59 (4.84–6.44)	5.13 ^#^ (4.42–5.96)	4.89 ^##^ (4.20–5.70)	5.19 ^#^ (4.48–6.02)	5.49 ^#^ (4.75–6.33)
	M+F	5.85 (5.30–6.45)	5.69 (5.15–6.29)	5.77 (5.22–6.37)	5.57 (5.03–6.16)	5.85 (5.30–6.46)	6.04 (5.48–6.66)

^#^ Hash marks statistically significant differences between male (M) and female (F) samples from the same sample group (values in the same column and adjacent row are statistically significantly different; ^#^ *p* < 0.01; ^##^ *p* < 0.001).

**Table 2:** Leukogram indicators in humans of both sexes. Mean values and 95% confidence interval of the three independent experimental groups (30 samples per group were exposed to 5G RF-EMR at frequency of 700 MHz, 2500 MHz, and 3500 MHz) and the control group.

		5G Radiofrequency Electromagnetic Radiation (5G RF-EMR) Frequencies					
		700 MHz		2500 MHz		3500 MHz	
Number of Humans		30	30	30	30	30	30
Group	Gender	Control	Experimental	Control	Experimental	Control	Experimental
Total leukocytes (10^9^/L)	M	6.82 (6.20–7.50)	6.85 (6.23–7.53)	6.45 (5.83–7.13)	6.31 (5.70–7.00)	6.28 (5.66–6.96)	6.22 (5.60–6.90)
	F	6.99 (6.37–7.67)	7.13 (6.51–7.81)	6.77 (6.15–7.45)	6.78 (6.17–7.47)	7.06 (6.44–7.74)	7.10 (6.48–7.78)
	M+F	6.91 (6.46–7.38)	6.99 (6.54–7.46)	6.61 (6.16–7.08)	6.54 (6.10–7.02)	6.66 (6.21–7.13)	6.64 (6.20–7.12)
Neutrophils (10^9^/L)	M	3.94 (3.43–4.63)	3.98 (3.47–4.67)	3.57 (3.07–4.27)	3.55 (3.04–4.25)	3.51 (3.00–4.21)	3.45 ^#^ (2.95–4.16)
	F	4.11 (3.60–4.80)	4.18 (3.67–4.86)	4.16 (3.64–4.84)	4.22 (3.71–4.90)	4.31 (3.79–4.99)	4.33 ^#^ (3.81–5.01)
	M+F	4.03 (3.65–4.49)	4.08 (3.70–4.54)	3.84 (3.46–4.32)	3.85 (3.47–4.33)	3.87 (3.48–4.35)	3.84 (3.45–4.32)
Basophils (10^9^/L)	M	0.031 (0.024–0.038)	0.031 (0.024–0.038)	0.031 ^#^ (0.024–0.039)	0.035 ^#^ (0.029–0.043)	0.035 (0.028–0.042)	0.035 (0.028–0.042)
	F	0.023 (0.016–0.030)	0.027 (0.020–0.034)	0.021 ^#^ (0.014–0.028)	0.023 ^#^ (0.016–0.031)	0.027 (0.020–0.034)	0.027 (0.021–0.035)
	M+F	0.027 (0.022–0.032)	0.029 (0.024–0.034)	0.026 (0.021–0.031)	0.029 (0.024–0.034)	0.031 (0.026–0.036)	0.031 (0.026–0.036)
Eosinophils (10^9^/L)	M	0.22 (0.16–0.29)	0.22 (0.16–0.29)	0.19 (0.14–0.27)	0.19 (0.14–0.27)	0.21 (0.15–0.28)	0.20 (0.15–0.28)
	F	0.13 (0.08–0.21)	0.13 (0.08–0.21)	0.10 (0.06–0.19)	0.11 (0.07–0.20)	0.11 (0.07–0.20)	0.11 (0.06–0.19)
	M+F	0.17 (0.13–0.22)	0.17 (0.13–0.23)	0.14 (0.10–0.20)	0.15 (0.11–0.21)	0.15 (0.11–0.21)	0.15 (0.10–0.20)
Lymphocytes (10^9^/L)	M	2.22 (1.95–2.49)	2.22 (1.94–2.49)	2.19 (1.91–2.46)	2.15 (1.87–2.42)	2.10 (1.83–2.38)	2.10 (1.83–2.37)
	F	2.33 (2.06–2.31)	2.39 (2.12–2.67)	1.95 * (1.67–2.22)	2.10 * (1.83–2.37)	2.24 (1.97–2.52)	2.25 (1.97–2.52)
	M+F	2.27 (2.08–2.47)	2.31 (2.11–2.50)	2.07 (1.87–2.26)	2.12 (1.93–2.32)	2.17 (1.98–2.37)	2.18 (1.98–2.37)
Monocytes (10^9^/L)	M	0.41 (0.35–0.49)	0.39 (0.33–0.48)	0.41 (0.35–0.49)	0.39 (0.33–0.48)	0.43 (0.36–0.51)	0.43 (0.37–0.51)
	F	0.39 (0.33–0.48)	0.39 (0.33–0.48)	0.34 (0.28–0.43)	0.33 (0.27–0.42)	0.37 (0.31–0.45)	0.39 (0.33–0.48)
	M+F	0.40 (0.35–0.46)	0.39 (0.35–0.45)	0.37 (0.33–0.43)	0.36 (0.31–0.42)	0.39 (0.35–0.45)	0.41 (0.36–0.47)

* Asterisks indicate statistically significant differences between control and experimental sample groups (values in the same row and adjacent column are statistically significantly different; * *p* < 0.05). ^#^ Hash marks statistically significant differences between male (M) and female (F) samples from the same sample group (values in the same column and adjacent row are statistically significantly different; ^#^ *p* < 0.01).

**Table 3:** Erythrogram indicators in humans of both sexes. Mean values and 95% confidence interval of three independent experimental groups (30 samples per group were exposed to 5G RF-EMR at frequency of 700 MHz, 2500 MHz, and 3500 MHz) and the control group.

		5G Radiofrequency Electromagnetic Radiation (5G RF-EMR) Frequencies					
		700 MHz		2500 MHz		3500 MHz	
Number of Humans		30	30	30	30	30	30
Group	SEX	Control	Experimental	Control	Experimental	Control	Experimental
Total erythrocytes (10^12^/L)	M	5.05 ^##^ (4.88–5.22)	5.06 ^##^ (4.89–5.23)	5.13 ^##^ (4.96–5.30)	5.11 ^##^ (4.93–5.28)	5.01 ^##^ (4.84–5.18)	5.04 ^##^ (4.87–5.21)
	F	4.54 ^##^ (4.37–4.71)	4.55 ^##^ (4.38–4.73)	4.48 ^##^ (4.31–4.65)	4.50 ^##^ (4.33–4.67)	4.48 ^##^ (4.31–4.65)	4.49 ^##^ (4.32–4.67)
	M+F	4.80 (4.67–4.92)	4.81 (4.68–4.93)	4.80 (4.68–4.92)	4.80 (4.68–4.92)	4.75 (4.63–4.87)	4.77 (4.65–4.89)
Reticulocytes (10^9^/L)	M	57.13 (48.03–67.96)	57.20 (48.10–68.03)	58.50 (48.40–70.70)	59.77 (50.01–71.43)	57.14 (47.75–68.38)	59.21 (49.79–70.42)
	F	48.47 (39.50–59.47)	47.80 (38.85–58.82)	53.58 (43.57–65.90)	54.25 (44.22–66.55)	43.47 (34.60–54.60)	46.47 (37.54–57.52)
	M+F	52.62 (46.02–60.18)	52.29 (45.68–59.86)	55.99 (48.66–64.42)	56.94 (49.72–65.21)	49.84 (43.10–57.62)	52.45 (45.72–60.18)
Reticulocytes (%)	M	11.23 (9.43–13.31)	11.22 (9.43–13.31)	11.32 (9.32–13.68)	11.65 (9.70–13.94)	11.40 (9.53–13.58)	11.73 (9.84–13.93)
	F	10.60 (8.86–12.64)	10.41 (8.64–12.44)	11.75 (9.72–14.14)	11.87 (9.82–14.27)	9.59 (7.93–11.55)	10.23 (8.52–12.25)
	M+F	10.91 (9.63–12.34)	10.81 (9.54–12.23)	11.53 (10.07–13.17)	11.76 (10.31–13.38)	10.46 (9.18–11.89)	10.96 (9.65–12.41)
Haematocrit (L/L)	M	0.46 ^##^ (0.45–0.48)	0.46 ^##^ (0.45–0.48)	0.47 ^##^ (0.45–0.49)	0.47 ^##^ (0.45–0.48)	0.46 ^##^ (0.44–0.48)	0.47 ^##^ (0.45–0.48)
	F	0.42 ^##^ (0.40–0.44)	0.42 ^##^ (0.41–0.44)	0.41 ^##^ (0.40–0.43)	0.42 ^##^ (0.40–0.43)	0.42 ^##^ (0.40–0.43)	0.42 ^##^ (0.40–0.43)
	M+F	0.44 (0.43–0.45)	0.44 (0.43–0.45)	0.44 (0.43–0.45)	0.44 (0.43–0.45)	0.44 (0.43–0.45)	0.44 (0.43–0.45)
Haemoglobin (g/L)	M	152 ^##^ (147–157)	151 ^##^ (146–156)	153 ^##^ (148–158)	153 ^##^ (148–158)	151 ^##^ (146–156)	151 ^##^ (146–156)
	F	136 ^##^ (131–141)	136 ^##^ (131–141)	134 ^##^ (129–139)	134 ^##^ (129–139)	134 ^##^ (129–139)	135 ^##^ (130–140)
	M+F	144 (140–147)	144 (140–147)	143 (140–147)	144 (140–147)	142 * (139–146)	143 * (139–146)

* Asterisks indicate statistically significant differences between control and experimental sample groups (values in the same row and adjacent column are statistically significantly different; * *p* < 0.05). ^#^ Hash marks statistically significant differences between male (M) and female (F) samples from the same sample group (values in the same column and adjacent row are statistically significantly different; ^##^ *p* < 0.0001).

**Table 4:** The distribution of erythrocytes by volume and erythrocyte constants in humans of both sexes. Mean values and 95% confidence interval of three independent experimental groups (30 samples per group were exposed to 5G RF-EMR at frequency of 700 MHz, 2500 MHz, and 3500 MHz) and the control group.

		5G Radiofrequency Electromagnetic Radiation (5G RF-EMR) Frequencies					
		700 MHz		2500 MHz		3500 MHz	
Number of Humans		30	30	30	30	30	30
Group	Gender	Control	Experimental	Control	Experimental	Control	Experimental
MCV (fL)	M	91.72 (89.22–94.09)	91.81 (89.30–94.18)	91.61 * (89.10–93.98)	89.69 * (87.18–92.06)	92.23 (89.73–94.61)	92.24 (89.74–94.61)
	F	92.80 (90.30–95.18)	92.79 (90.28–95.16)	92.74 (90.24–95.12)	92.82 (90.32–95.20)	93.35 (90.85–95.73)	93.34 (90.84–95.72)
	M+F	92.26 (90.51–93.95)	92.30 (90.54–94.00)	92.18 (90.42–93.88)	91.28 (89.52–92.97)	92.80 (91.04–94.49)	92.80 (91.04–94.49)
MCH (pg)	M	30.02 (2932–30.70)	29.95 (29.25–30.63)	29.81 (29.12–30.49)	29.97 (29.27–30.65)	30.02 (29.32–30.70)	29.97 (29.27–30.65)
	F	29.91 (29.21–30.59)	29.87 (29.17–30.55)	29.95 (29.25–30.63)	29.88 (29.18–30.56)	29.99 (29.29–30.67)	30.00 (29.30–30.68)
	M+F	29.96 (29.47–30.45)	29.91 (29.42–30.39)	29.88 (29.39–30.37)	29.92 (29.43–30.41)	30.00 (29.51–30.49)	29.98 (29.49–30.47)
MCHC (g/L)	M	327 ^#^ (324–330)	326 ^#^ (323–329)	325 (322–328)	327 ^#^ (324–330)	326 ^#^ (323–329)	325 (322–328)
	F	322 ^#^ (319–325)	322 ^#^ (319–325)	323 (320–326)	322 ^#^ (319–325)	321 ^#^ (318–324)	321 (319–324)
	M+F	325 (323–327)	324 (322–326)	324 (322–326)	325 (322–327)	324 (321–326)	323 (321–325)
RDW (%)	M	12.98 (12.69–13.28)	12.97 (12.68–13.26)	13.03 (12.74–13.33)	13.02 (12.73–13.32)	13.07 (12.78–13.36)	13.06 (12.77–13.36)
	F	13.21 (12.92–13.51)	13.18 (12.89–13.48)	13.22 (12.93–13.52)	13.25 (12.95–13.54)	13.19 (12.90–13.49)	13.21 (12.92–13.51)
	M+F	13.10 (12.89–13.31)	13.07 (12.89–13.28)	13.13 (12.92–13.34)	13.13 (12.93–13.34)	13.13 (12.92–13.34)	13.14 (12.93–13.35)

MCV (mean cell volume)—average volume of erythrocytes; MCH (mean cell haemoglobin)—average haemoglobin concentration in erythrocytes; MCHC (mean cell haemoglobin concentration)—average haemoglobin concentration in one litre of erythrocytes; RDW (red cell distribution width)—distribution of erythrocytes by volume. * Asterisks indicate statistically significant differences between control and experimental sample groups (values in the same row and adjacent column are statistically significantly different; * *p* < 0.05). ^#^ Hash marks statistically significant differences between male (M) and female (F) samples from the same sample group (values in the same column and adjacent row are statistically significantly different; ^#^ *p* < 0.05).

**Table 5:** Thrombogram indicators in humans of both sexes. Mean values and 95% confidence interval of three independent experimental groups (30 samples per group were exposed to 5G RF-EMR at frequency of 700 MHz, 2500 MHz, and 3500 MHz) and the control group.

		5G Radiofrequency Electromagnetic Radiation (5G RF-EMR) Frequencies					
		700 MHz		2500 MHz		3500 MHz	
Number of Humans		30	30	30	30	30	30
Group	Gender	Control	Experimental	Control	Experimental	Control	Experimental
Platelets (10^9^/L)	M	257 (226–288)	263 (232–294)	259 (228–290)	259 (227–290)	246 ^##^ (215–277)	250 ^#^ (219–281)
	F	298 (267–329)	304 (272–335)	296 (265–327)	294 (263–326)	308 ^##^ (277–339)	304 ^#^ (273–335)
	M+F	277 (255–299)	283 (261–305)	277 (255–291)	277 (255–298)	277 (255–299)	277 (255–299)
MPV (fL)	M	8.55 (8.00–9.23)	8.52 (7.97–9.19)	8.75 (8.26–9.35)	8.75 (8.26–9.35)	8.84 (8.34–9.44)	8.89 (8.40–9.49)
	F	8.32 (7.75–9.03)	8.23 (7.66–8.94)	8.73 (8.23–9.32)	8.63 (8.14–9.23)	8.69 (8.19–9.28) *	8.52 (8.02–9.11) *
	M+F	8.43 (8.03–8.91)	8.37 (7.96–8.84)	8.74 (8.38–9.15)	8.69 (8.33–9.10)	8.76 (8.40–9.17)	8.70 (8.34–9.11)
Platelet activation (%)	M	0.002 (0.001–0.003)	0.002 (0.001–0.003)	0.002 (0.001–0.003)	0.002 (0.001–0.003)	0.002 (0.001–0.003)	0.002 (0.001–0.003)
	F	0.001 (0.001–0.002)	0.002 (0.001–0.003)	0.002 (0.001–0.003)	0.001 (0.001–0.002)	0.002 (0.001–0.003)	0.001 (0.001–0.002)
	M+F	0.002 (0.001–0.002)	0.002 (0.001–0.002)	0.002 (0.001–0.002)	0.002 (0.001–0.002)	0.002 (0.001–0.002)	0.002 (0.001–0.002)

MPV—mean platelet volume. * Asterisks indicate statistically significant differences between control and experimental sample groups (values in the same row and adjacent column are statistically significantly different; * *p* < 0.05). ^#^ Hash marks statistically significant differences between male (M) and female (F) samples from the same sample group (values in the same column and adjacent row are statistically significantly different; ^#^ *p* < 0.001; ^##^ *p* < 0.0001).

**Table 6:** Morphometric size indicators of human erythrocytes of both sexes. Mean values and 95% confidence interval of three independent experimental groups (30 samples per group were exposed to 5G RF-EMR at frequency of 700 MHz, 2500 MHz, and 3500 MHz) and control group.

			5G Radiofrequency Electromagnetic Radiation (5G RF-EMR) Frequencies					
			700 MHz		2500 MHz		3500 MHz	
Number of Humans			30	30	30	30	30	30
Group		Gender	Control	Experimental	Control	Experimental	Control	Experimental
Erythrocyte size indicators	Area (μm^2^)	M	44.51 ^###^* (44.26–44.77)	45.16 ^###^* (44.91–45.41)	45.11 (44.85–45.38)	45.45 (45.19–45.71)	45.28 ^###^ (45.04–45.53)	45.68 ^###^ (45.43–45.93)
		F	45.53 ^###^*** (45.27–45.79)	46.52 ^###^*** (46.26–46.78)	45.59 (45.33–45.84)	45.79 (45.53–46.04)	47.96 ^###^ (47.70–48.20)	48.03 ^###^ (47.77–48.28)
		M+F	45.02 *** (44.84–45.20)	45.84*** (45.66–46.02)	45.35 (45.17–45.53)	45.62 (45.43–45.80)	46.62 (46.44–46.79)	46.86 (46.68–47.03)
	Outline (μm)	M	26.67 ^###^*** (26.56–26.78)	27.16 ^###^*** (27.05–27.27)	26.68 ^##^ (26.57–26.80)	26.87 ^###^ (26.75–26.98)	27.03 ^###^ (26.92–27.14)	27.08 ^###^ (26.97–27.19)
		F	27.07 ^###^*** (26.96–27.19)	27.55 ^###^*** (27.43–27.66)	27.02 ^##^ (26.91–27.13)	27.28 ^###^ (27.16–27.39)	28.05 ^###^*** (27.94–28.16)	28.52 ^###^*** (28.41–28.63)
		M+F	26.87 *** (26.79–26.95)	27.35 *** (27.27–27.43)	26.85 ** (26.77–26.93)	27.07 ** (26.99–27.15)	27.54 *** (27.46–27.62)	27.80 *** (27.72–27.88)
	Minimum radius (μm)	M	3.43 ^###^** (3.42–3.44)	3.46 ^###^** (3.45–3.47)	3.47 (3.46–3.49)	3.48 (3.46–3.49)	3.47 ^###^ (3.46–3.48)	3.48 ^###^ (3.47–3.49)
		F	3.48 ^###^*** (3.47–3.49)	3.52 ^###^*** (3.51–3.53)	3.48 (3.47–3.49)	3.47 (3.46–3.48)	3.56 ^###^ (3.55–3.57)	3.58 ^###^ (3.56–3.59)
		M+F	3.45 *** (3.45–3.46)	3.49 *** (3.48–3.50)	3.48 (3.47–3.48)	3.47 (3.47–3.48)	3.52 (3.51–3.52)	3.53 (3.52–3.54)
	Maximum radius (μm)	M	4.04 ^##^* (4.03–4.05)	4.07 ^###^* (4.06–4.08)	4.05 ^#^ (4.03–4.06)	4.07 ^#^ (4.06–4.09)	4.07 ^###^ (4.06–4.09)	4.09 ^###^ (4.08–4.11)
		F	4.08 ^##^*** (4.06–409)	4.13 ^###^*** (4.11–4.14)	4.09 ^#^ (4.07–4.10)	4.11 ^#^ (4.10–4.12)	4.20 ^###^ (4.19–421)	4.19 ^###^ (4.18–4.20)
		M+F	4.06 *** (4.05–4.07)	4.10 *** (4.09–4.11)	4.07 ** (4.06–4.08)	4.09 ** (4.08–4.10)	4.14 (4.13–4.15)	4.14 (4.13–4.15)
	Convex area (μm^2^)	M	44.99 ^###^* (44.73–45.25)	45.68 ^###^* (45.42–45.94)	45.57 (45.30–45.83)	45.91 (45.65–46.18)	45.78 ^###^ (45.53–46.03)	46.18 ^###^ (45.93–46.44)
		F	46.02 ^###^*** (45.76–46.28)	47.05 ^###^*** (46.78–47.31)	46.07 (45.81–46.33)	45.30 (46.04–46.52)	48.50 ^###^ (48.25–48.76)	48.64 ^###^ (48.38–48.90)
		M+F	45.50 *** (45.32–45.69)	46.36 *** (46.18–46.55)	45.82 (45.63–46.00)	46.11 (45.92–46.29)	47.14 (46.96–47.32)	47.41 (47.23–47.59)
	Length (µm)	M	7.93 ^##^* (7.90–7.95)	7.99 ^###^* (7.97–8.02)	7.96 ^##^ (7.93–7.99)	8.00 ^#^ (7.97–8.02)	8.00 ^###^ (7.99–8.03)	8.05 ^###^ (8.03–8.08)
		F	8.01 ^##^*** (7.98–8.03)	8.10 ^###^*** (8.07–8.13)	8.04 ^##^ (8.01–8.06)	8.07 ^#^ (8.04–8.09)	8.25 ^###^ (8.23–8.28)	8.23 ^###^ (8.20–8.26)
		M+F	7.97 *** (7.95–7.99)	8.05 *** (8.03–8.06)	7.99 (7.98–8.02)	8.03 (8.01–8.05)	8.13 (8.11–8.15)	8.14 (8.12–8.16)
	Width (µm)	M	7.18 ^###^** (7.15–7.20)	7.24 ^###^** (7.22–7.25)	7.25 (7.22–7.27)	7.29 (7.24–7.29)	7.25 ^###^ (7.22–7.27)	7.26 ^###^ (7.24–7.28)
		F	7.27 ^###^*** (7.25–7.30)	7.37 ^###^*** (7.34–7.39)	7.25 (7.23–7.27)	7.26 (7.24–7.29)	7.44 ^###^ (7.42–7.47)	7.48 ^###^ (7.46–7.51)
		M+F	7.22 *** (7.21–7.24)	7.30 *** (7.29–7.32)	7.25 (7.23–7.26)	7.26 (7.25–7.28)	7.34 (7.33–7.36)	7.37 (7.36–7.39)

* Asterisks indicate statistically significant differences between control and experimental sample groups (values in the same row and adjacent column are statistically significantly different; * *p* < 0.05; ** *p* < 0.001; *** *p* < 0.0001). ^#^ Hash marks statistically significant differences between male (M) and female (F) samples from the same sample group (values in the same column and adjacent row are statistically significantly different; ^#^ *p* < 0.05; ^##^ *p* < 0.001; ^###^ *p* < 0.0001).

**Table 7:** Morphometric shape indicators of human erythrocytes of both sexes. Mean values and 95% confidence interval of three independent experimental groups (30 samples per group were exposed to 5G RF-EMR at frequency of 700 MHz, 2500 MHz and 3500 MHz) and control group.

			5G Radiofrequency Electromagnetic Radiation (5G RF-EMR) Frequencies					
			700 MHz		2500 MHz		3500 MHz	
Number of Humans			30	30	30	30	30	30
Group		Gender	Control	Experimental	Control	Experimental	Control	Experimental
Morphometric shape indicators of erythrocytes	Contour index	M	4.00 *** (3.99–4.01)	4.05 *** (4.04–4.06)	3.98 ^##^ (3.97–3.99)	3.99 ^###^ (3.98–4.00)	4.02 ^###^ (4.01–4.03)	4.01 ^###^ (4.00–4.02)
		F	4.02 * (4.01–4.03)	4.04 * (4.03–4.05)	4.01 ^##^** (4.00–4.02)	4.04 ^###^** (4.03–4.05)	4.06 ^###^*** (4.05–4.06)	4.12 ^###^*** (4.11–4.13)
		M+F	4.01*** (4.00–4.02)	4.04 *** (4.04–4.05)	3.99 *** (3.96–4.00)	4.01 *** (4.01–4.02)	4.04 *** (4.03–4.04)	4.07 *** (4.06–407)
	Solidity	M	0.9895 *** (0.9893–0.9897)	0.9887 ^###^*** (0.9885–0.9889)	0.9901 (0.9899–0.9903)	0.9899 ^###^ (0.9897–0.9901)	0.9893 ^##^ (0.9891–0.9895)	0.9893 ^###^ (0.9891–0.9895)
		F	0.9894 * (0.9892–0.9896)	0.9889 ^###^* (0.9887–0.9891)	0.9896 ** (0.9894–0.9898)	0.9890 ^###^** (0.9888–0.9892)	0.9887 ^##^*** (0.9885–0.9889)	0.9875 ^###^*** (0.9873–0.9877)
		M+F	0.9894 *** (0.9893–0.9896)	0.9888 *** (0.9887–0.9889)	0.9899 ** (0.9897–0.9900)	0.9895 ** (0.9893–0.9896)	0.9890 *** (0.9889–0.9891)	0.9884 *** (0.9883–0.9885)
	Roundness	M	0.868 (0.866–0.871)	0.867 (0.864–0.869)	0.875 ^#^ (0.872–0.878)	0.871 ^##^ (0.869–0.874)	0.868 (0.865–0.870)	0.867 (0.864–0.869)
		F	0.871 (0.869–0.874)	0.870 (0.867–0.872)	0.868 ^#^ (0.866–0.871)	0.863 ^##^ (0.861–0.866)	0.864 (0.862–0.867)	0.870 (0.867–0.872)
		M+F	0.870 (0.868–0.871)	0.868 (0.866–0.870)	0.872 ** (0.870–0.873)	0.867 ** (0.865–0.869)	0.866 (0.864–0.868)	0.868 (0.867–0.870)
	Ellipticity	M	1.11 (1.10–1.11)	1.10 (1.10–1.11)	1.10 ^##^ (1.10–1.10)	1.10 ^##^ (1.10–1.11)	1.11 (1.11–1.12)	1.11 ^##^ (1.11–1.12)
		F	1.10 (1.10–1.11)	1.10 (1.10–1.10)	1.11 ^##^ (1.10–1.11)	1.11 ^##^ (1.11–1.12)	1.11 ** (1.11–1.12)	1.10 ^##^** (1.10–1.11)
		M+F	1.11 (1.10–1.11)	1.11 (1.10–1.11)	1.11 (1.11–1.11)	1.11 (1.11–1.11)	1.11 (1.11–1.11)	1.11 (1.11–1.11)
	Elongation	M	0.049 (0.048–0.051)	0.049 (0.047–0.050)	0.046 ^##^ (0.045–0.048)	0.048 ^##^ (0.046–0.049)	0.049 (0.048–0.050)	0.051 ^##^ (0.050–0.053)
		F	0.048 (0.046–0.049)	0.047 (0.045–0.048)	0.051 ^##^ (0.050–0.053)	0.052 ^##^ (0.050–0.053)	0.051 * (0.050–0.052)	0.047 ^##^* (0.045–0.048)
		M+F	0.048 (0.047–0.049)	0.048 (0.047–0.0549	0.049 (0.048–0.050)	0.050 (0.049–0.051)	0.050 (0.049–0.051)	0.049 (0.048–0.050)
	Form factor	M	0.788 ^##^*** (0.785–0.791)	0.773 ^###^*** (0.770–0.776)	0.80 ^##^ (0.79–0.80)	0.793 ^###^ (0.790–0.796)	0.782 ^###^ (0.780–0.785)	0.784 ^###^ (0.782–0.787)
		F	0.783 ^##^** (0.780–0.786)	0.775 ^###^** (0.772–0.778)	0.788 ^##^*** (0.785–0.790)	0.778 ^###^*** (0.775–0.781)	0.768 ^###^*** (0.765–0.771)	0.750 ^###^*** (0.747–0.752)
		M+F	0.785 *** (0.783–0.787)	0.774 *** (0.772–0.776)	0.793 *** (0.791–0.794)	0.785 *** (0.783–0.787)	0.775 *** (0.773–0.777)	0.767 *** (0.765–0.769)

Ellipticity = length/width; elongation = (length − width)/(length + width); solidity = area/convex area; roundness = (4 × area)/[π × (maximum radius)^2^]; form factor = 4π × area/outline^2^; contour index = outline/√area. * Asterisks indicate statistically significant differences between control and experimental sample groups (values in the same row and adjacent column are statistically significantly different; * *p* < 0.05; ** *p* < 0.001; *** *p* < 0.0001). ^#^ Hash marks statistically significant differences between male (M) and female (F) samples from the same sample group (values in the same column and adjacent row are statistically significantly different; ^#^ *p* < 0.05; ^##^ *p* < 0.001; ^###^ *p* < 0.0001).

**Table 8:** Eigenvalues of individual morphometric size and shape indicators of erythrocytes for the three principal components (factors) of the principal component analysis. Three factors (1, 2, 3) with a characteristic root λ ≥ 1 were retained—Kaiser criterion.

Morphometric Size and Shape Indicators of Erythrocytes	Erythrocyte Size	Erythrocyte Form	
	Factor 1	Factor 2	Factor 3
Outline (µm)	* 0.97		
Convexity (µm^2^)	0.94		
Area (µm^2^)	0.2		
Length (µm)	0.88		
Width (µm)	0.80		
Roundness		0.91	
Form factor			0.70
Contour index			−0.74
Elongation		* −0.95	
Ellipticity		−0.94	
Solidity			* 0.77
Characteristic root (λ) andexplained variance (%)	6.57 (50.6)	3.71 (28.6)	2.32 (17.8)
Cumulative variance (%)	50.6	79.2	97.0

Ellipticity = length/width; elongation = (length − width)/(length + width); solidity = area/convex area; roundness = (4 × area)/[π × (maximum radius)^2^]; form factor = 4π × area/outline^2^; contour index = outline/√area. * Asterisks mark the most important value of erythrocytes for each factor (values greater than 0.60 are shown for each factor).

**Table 9:** Erythrocyte subpopulations (ESs) based on the most important erythrocyte values for each factor (data expressed as mean and standard deviation).

Erythrocyte Subpopulations		Erythrocyte Size					
	N (%)	Outline (µm)	Convex Area (µm^2^)	Area (µm^2^)	Length (µm)	Width (µm)	
ES 1	17,592 (67.5)	25.9 ± 1.71	43.0 ± 4.10	42.6 ± 4.04	7.76 ± 0.46	7.03 ± 0.42	
ES 2	8476 (32.5)	29.9 ± 2.53	53.4 ± 3.63	52.7 ± 3.55	8.66 ± 0.41	7.83 ± 0.37	
			Erythrocyte form				
		Roundness	Ellipticity	Elongation	Solidity	Form factor	Contour index
ES 1	17,592 (675)	0.871 ± 0.059	1.106 ± 0.078	0.049 ± 0.033	0.989 ± 0.003	0.795 ± 0.049	3.98 ± 0.148
ES 2	8476 (32.5)	0.863 ± 0.059	1.107 ± 0.074	0.049 ± 0.032	0.987 ± 0.006	0.749 ± 0.085	4.12 ± 0.305

Ellipticity = length/width; elongation = (length − width)/(length + width); solidity = area/convex area; roundness = (4 × area)/[π × (maximum radius)^2^]; form factor = 4π × area/outline^2^; contour index = outline/√area. ES 1—subpopulation of smaller and rounder erythrocytes with more regular/smoother membranes; ES 2—subpopulation of larger erythrocytes with more irregular membranes.

## Data Availability

The data presented in this study are available on reasonable request from the corresponding author. The data are not publicly available due to planned research in the future.
